# Genomic Epidemiology of Human Respiratory Syncytial Virus, Minnesota, USA, July 2023–February 2024

**DOI:** 10.3201/eid3011.241000

**Published:** 2024-11

**Authors:** Daniel Evans, Henry Kunerth, Erica Mumm, Sarah Namugenyi, Matthew Plumb, Sarah Bistodeau, Scott A. Cunningham, Bryan Schmitt, Karen Martin, Katherine Como-Sabetti, Ruth Lynfield, Xiong Wang

**Affiliations:** Health Protection Bureau, Minnesota Department of Health, St. Paul, Minnesota, USA (D. Evans, H. Kunerth, E. Mumm, S. Namugenyi, M. Plumb, S. Bistodeau, S.A. Cunningham, K. Martin, K. Como-Sabetti, R. Lynfield, X. Wang); Children’s Minnesota Hospital, Minneapolis, Minnesota, USA (B. Schmitt)

**Keywords:** Respiratory syncytial virus, genomic epidemiology, viral surveillance, Minnesota, viruses, respiratory infections

## Abstract

We recently expanded the viral genomic surveillance program in Minnesota, USA, to include human respiratory syncytial virus. We performed whole-genome sequencing of 575 specimens collected at Minnesota healthcare facilities during July 2023–February 2024. Subgroups A and B differed in their genomic landscapes, and we identified 23 clusters of genetically identical genomes.

Human respiratory syncytial virus (RSV) is a major respiratory pathogen with increased risk of severe infections among infants and young children, elderly persons, and persons with underlying health conditions, including immunocompromise (*1*). Investigators have applied whole-genome sequencing (WGS) for retrospective RSV surveillance and outbreak investigation in the United States (*2*), but WGS has not yet been documented as a tool for prospective surveillance. Although researchers have developed and improved several genetic typing schemes to facilitate more granular characterization of RSV (*3*), application of those schemes in genomic epidemiology has not been thoroughly evaluated. We report preliminary findings from our recently established genomic surveillance program for RSV in Minnesota, USA. Because this study was conducted as a component of public health surveillance subject to Minnesota Reporting Rules, our investigation required no institutional review board approval.

## The Study

During July 2023–February 2024, we collected RSV-positive nasal or nasopharyngeal specimens submitted voluntarily from outpatients and inpatients tested by quantitative reverse transcription PCR (qRT-PCR) or rapid antigen detection assays in 11 healthcare facilities in Minnesota. Specimens arrived with limited patient data (name, sex assigned at birth, date of birth, date of specimen collection, and outpatient or inpatient status). 

We amplified genomes from specimens using 50 pairs of PCR primers that generated overlapping amplicons, as described by Maloney et al. (unpub. data, https://virological.org/t/preliminary-results-from-two-novel-artic-style-amplicon-based-sequencing-approaches-for-rsv-a-and-rsv-b/918), and sequenced genomes using the GridION platform with R9 flow cell chemistry (Oxford Nanopore Technologies, https://nanoporetech.com). We performed genome assembly, quality control, and viral subtyping using the nf-core Viralrecon pipeline (*4*), using a quality threshold of 20-fold coverage over 90% of the viral genome. We used Nextclade software to subgroup genomes based on the whole-genome lineage typing scheme described by Goya et al. (*3*) as available through Nextclade in June 2024 (*5*).

We successfully sequenced 575 RSV genomes from this cohort of case-patients. Median patient age was 2 years; 91.8% were <18 years of age, and 5% were ≥65 years of age. Patient sex was 53.4% male and 46.6% female. We classified 287 (49.9%) genomes as subgroup A and 288 (50.1%) as subgroup B (Table 1). Most RSV-A genomes (98.9%; n = 284) were distributed among 4 whole-genome lineages: A.D.1 (10.8%; n = 31), A.D.3 (18.5%; n = 53), A.D.5 (38.0%; n = 109), and A.D.5.2 (31.7%; n = 91). By contrast, most RSV-B genomes (95.1%; n = 274) belonged to a single lineage, B.D.E.1.

**Table 1:** Whole-genome sequencing results for RSV-A and RSV-B specimens collected from patients in Minnesota, USA, July 2023–February 2024*

Whole-genome lineage	No. (% of subgroup)	Cases in RSV-NET (% of WGS lineage)	Twin Cities metro (% of WGS lineage)	Mean pairwise phylogenetic distance	Earliest specimen collection, mo	Estimated time to most recent common ancestor (95% CI)
A.D.1	31 (10.8)	2 (6.5)	26 (83.9)	0.00555	2023 Sep	Jun 2017 (Jan 1996–Jan 2021)
A.D.3	53 (18.5)	5 (9.4)	40 (75.5)	0.00718	2023 Aug	Aug 2017 (Jun 1996–Jan 2021)
A.D.3.1	1 (0.3)	0	1 (100)	NA	2024 Jan	NA
A.D.5	109 (38.0)	25 (22.9)	103 (94.5)	0.00076	2023 Aug	Aug 2018 (Mar 2002–Nov 2021)
A.D.5.1	1 (0.3)	0	1 (100)	NA	2023 Dec	NA
A.D.5.2	91 (31.7)	32 (35.1)	74 (81.3)	0.00252	2023 Oct	Apr 2021 (May 2015–Oct 2022)
A.D.5.3	1 (0.3)	0	NA (unknown)	NA	2023 Sep	NA
B.D.4.1.1	12 (4.2)	4 (33.3)	12 (100)	0.00513	2023 Oct	Mar 2015 (Jun 1976–May 2019)
B.D.E.1	274 (95.1)	49 (17.9)	189 (69)	0.00384	2023 Jul	Oct 2019 (Nov 2000–Jul 2021)
B.D.E.2	2 (0.7)	0	2 (100)	NA	2023 Jul	NA

*Counts and percentages exclude cases with out-of-state or of unknown residence. Pairwise phylogenetic distances and estimated times of most recent common ancestors were calculated using Minnesota HRSV genomes only for lineages with >3 genomes. NA, not applicable; RSV, respiratory syncytial virus; RSV-NET, Respiratory Syncytial Virus Hospitalization Surveillance Network; Twin Cities Metro, Minneapolis-St Paul metropolitan area; WGS, whole-genome sequencing.

**Figure 1:**
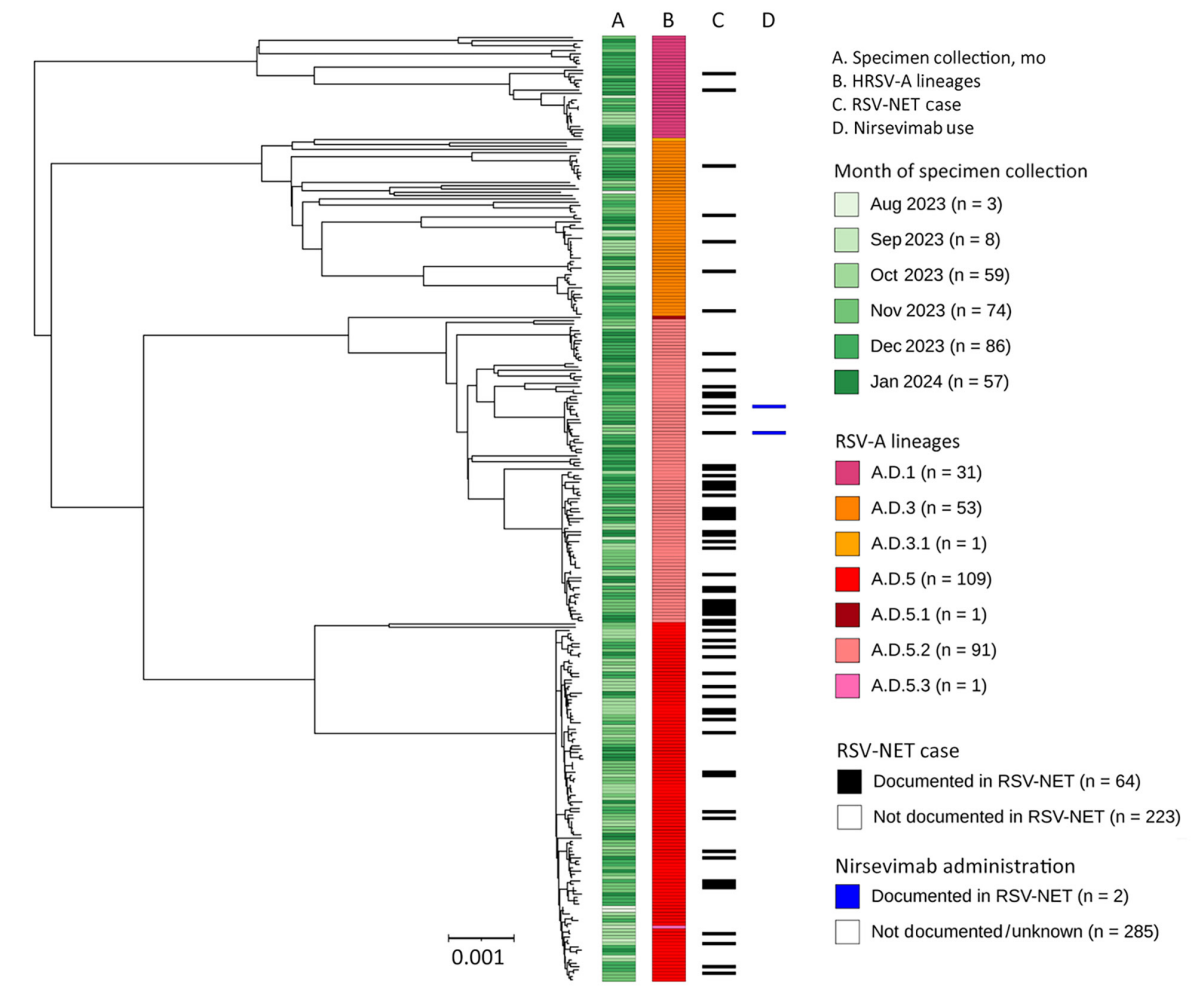
Midpoint-rooted, time-scaled phylogenetic tree of RSV-A whole-genome sequences, Minnesota, USA, October 2023–January 2024. Tree was generated using Interactive Tree of Life (https://itol.embl.de) software. Column annotations denote (from left to right) month of specimen collection, whole-genome lineage classification, documentation of the infected case in the RSV-NET surveillance database for RSV-associated hospitalizations, and administration of nirsevimab before infection as documented in RSV-NET. Scale bar indicates estimated substitution rate calculated from inputs of time and phylogenetic distance. RSV, respiratory syncytial virus; RSV-NET, Respiratory Syncytial Virus Hospitalization Surveillance Network.

**Figure 2:**
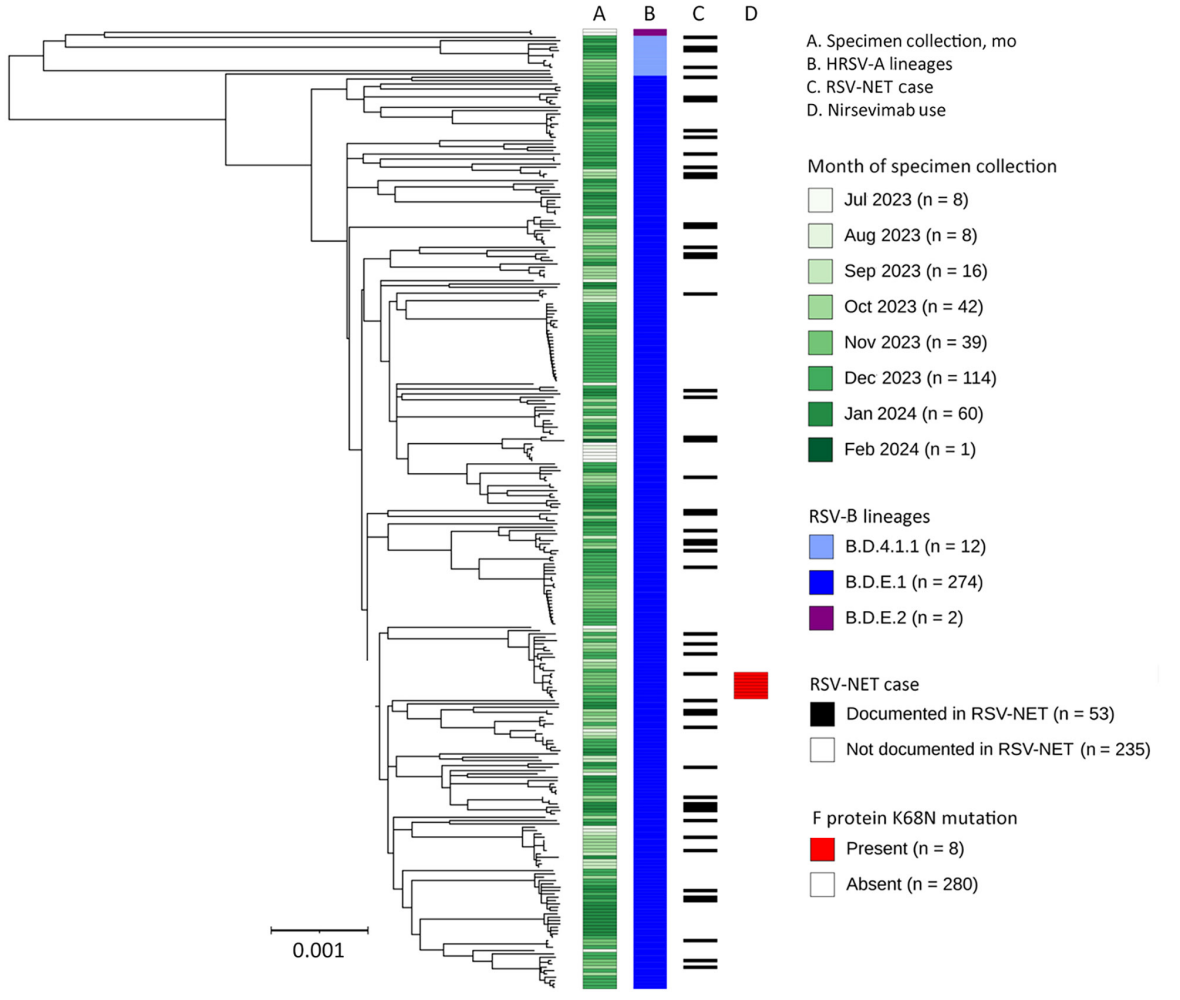
Midpoint-rooted, time-scaled phylogenetic tree of RSV-B whole-genome sequences, Minnesota, USA, October 2023–January 2024. Tree was generated using Interactive Tree of Life (https://itol.embl.de) software. Column annotations denote (from left to right) month of specimen collection, whole-genome lineage classification, documentation of the infected case in the RSV-NET surveillance database for RSV-associated hospitalizations, and the predicted nirsevimab resistance amino acid mutation K68N in the F protein gene sequence. Scale bar indicates estimated substitution rate calculated from inputs of time and phylogenetic distance. RSV, human respiratory syncytial virus; RSV-NET, Respiratory Syncytial Virus Hospitalization Surveillance Network.

We constructed phylogenetic trees using Nextstrain’s Augur pipeline version 24.3.0 (Figures 1, 2) (*6*,*7*). We aligned viral genome assemblies to Nextstrain’s default reference sequences (hRSV/A/England/397/2017 and hRSV/B/Australia/VIC-RCH056/2019) by using MAFFT version 7.526 (*8*) and then constructed and refined distance-scaled and time-scaled trees from those alignments by using IQTree version 2.3.3 and TimeTree version 0.11.3 (*9*,*10*). Comparisons of tree architecture showed greater diversity among all RSV-A genomes (mean pairwise p-distance 0.00933) than among all RSV-B genomes (mean pairwise p-distance 0.00455) (*11*). We observed pairwise p-distances among lineages with >3 genomes to be more comparable across subgroups (Table 1). Time-scaled phylogenetic analyses specific to the genomes we sequenced estimated the divergence of lineages to have occurred between 2 and 8 years before their earliest specimen collection dates (Table 1; Appendix Figure). The 95% CIs excluded divergence dates that were more recent than 1–5 years before earliest specimen collection.

We identified single-nucleotide polymorphisms (SNPs) from reference-based whole-genome alignments using SNP-dists version 0.8.2 (*12*). Pairwise comparisons showed that 32.3% of genomes were identical to >1 other genome at 0 SNPs (RSV-A, 33.7%, n = 97; HRSV-B, 30.9%, n = 89) and 53% within 1 SNP (RSV-A, 54%, n = 155; RSV-B, 52.1%, n = 150). We found 23 clusters of at least 3 genomes with shared nucleotide identity at 0 SNPs (RSV-A, n = 14; RSV-B, n = 9). Those clusters included 19.5% of all genomes (RSV-A, 20.6%, n = 59; RSV-B, 18.4%, n = 53). Additionally, we identified 1 clade of 8 RSV-B genomes with the F protein amino acid mutation K68N, which is associated with nirsevimab resistance (*13*). This clade had an estimated time of most recent common ancestor of September–November 2023 (Figure 2).

To assess our ability to link RSV genomes to known severe infections, we cross-referenced the names and birthdates of case patients whose specimens we sequenced against the Respiratory Syncytial Virus Hospitalization Surveillance Network (RSV-NET). RSV-NET is a component of the Centers for Disease Control and Prevention Emerging Infections Program focused on sentinel population-based surveillance of RSV-associated hospitalizations and deaths (Table 2; Figure 1) (*14*). Among 531 genomes collected during October 2023–January 2024, the peak months of specimen collection, 117 (22%) were noted among RSV-NET cases (RSV-A, 55.2%, n = 64; RSV-B, 45.3%, n = 53). Those 117 genomes represented 6.3% of all hospitalized cases of RSV during that period. Nine (39.1%) of the 23 clusters we identified included >1 RSV-NET case (RSV-A, 50%, n = 7; RSV-B, 22.2%, n = 2), and 13.4% of clustered cases were in RSV-NET (RSV-A, 20.3%, n = 2; RSV-B, 5.7%, n = 3). Two RSV-NET case-patients with sequenced RSV—both with A.D.5.2 infections—were documented to have received nirsevimab (Figure 1). Of the 8 RSV-B cases whose genomes carried the K68N F protein mutation, 1 was documented in RSV-NET (Figure 2).

**Table 2:** Comparison of RSV cases with WGS data to hospitalized or deceased case patients documented in RSV-NET, Minnesota, USA, October 2023–January 2024*

Parameter	RSV-NET cases, Oct 2023–Jan 2024	RSV cases with WGS data, Oct 2023–Jan 2024	RSV-NET cases with WGS data, Oct 2023–Jan 2024
No.	1,847	524	116
Median age, y	2	1.7	1
Sex, %			
F	51.1	49.2	51.7
M	48.9	50.8	48.3
White race, %	63.6	45.2	53.5
Minneapolis-St. Paul metropolitan area, %	65.0	75.2	87.1
Total RSV-NET cases, %	NA	NA	6.3
Sequenced cases, %	NA	NA	20.2

*NA, not applicable; RSV-NET, RSV, respiratory syncytial virus; Respiratory Syncytial Virus Hospitalization Surveillance Network; WGS, whole-genome sequencing.

To assess potential biases in our sampling for WGS, we performed a preliminary representativeness analysis of hospitalized RSV case-patients with or without collected WGS data. The cohort of sequenced cases documented in RSV-NET was not fully representative of all RSV-NET cases (Table 2). Compared with all RSV-NET case-patients, RSV-NET case-patients with sequenced RSV tended to be younger (median age 2 years for all RSV-NET vs. 1 year for sequenced RSV-NET; p = 0.0193), included a smaller proportion of cases of White versus non-White race (63.6% of all RSV-NET vs. 53.5% for sequenced RSV-NET; p = 0.0014), and were more likely to live within the Minneapolis-St. Paul metropolitan area (65% of all RSV-NET vs. 87.1% of sequenced RSV-NET; p<0.001).

## Conclusions

Our findings from a new statewide genomic surveillance program of RSV infections in Minnesota, USA, show that applying WGS for RSV surveillance can yield insights into viral circulation and population dynamics. Prospective sequencing revealed differing genomic landscapes of subgroups A and B and contextualized their genetic diversity within the state. The detection of clusters within the viral population shows the potential use of WGS for detection and investigation of RSV outbreaks at state or local levels. Our study also provided novel cross-referencing of viral genomic data against sentinel clinical surveillance of severe RSV infections.

Our study’s skewed and localized convenience sampling limited the analytical potential of our findings. The limited geographic and temporal scope of our study also potentially introduced variability in our time-scaled analyses and location-specific discrepancies between RSV evolution in Minnesota and in other regions. In future work, we will expand the collection of genomic and epidemiologic data with more targeted collection of specimens, to improve the scope and representativeness of our sampling approach such that these questions can be investigated more thoroughly. We also intend to perform sufficiently powered epidemiologic analyses on the emergence of mutations linked to vaccine escape, virulence, and transmissibility. However, our results demonstrate that prospective genomic surveillance of RSV can identify the emergence of mutations and clades of epidemiologic significance, such as those linked to evolution of vaccines or monoclonal antibody resistance (*2**,**3*). 

Appendix Additional information for genomic epidemiology of human respiratory syncytial virus, Minnesota, USA, July 2023–February 2024.

## References

[1] Shang Z, Tan S, Ma D. Respiratory syncytial virus: from pathogenesis to potential therapeutic strategies. Int J Biol Sci. 2021;17:4073–91. 10.7150/ijbs.64762 34671221 PMC8495404

[2] Goya S, Sereewit J, Pfalmer D, Nguyen TV, Bakhash SAKM, Sobolik EB, et al. Genomic characterization of respiratory syncytial virus during 2022–23 outbreak, Washington, USA. Emerg Infect Dis. 2023;29:865–8. 10.3201/eid2904.221834 36878012 PMC10045680

[3] Goya S, Ruis C, Neher RA, Meijer A, Aziz A, Hinrichs AS, et al. Standardized phylogenetic classification of human respiratory syncytial virus below the subgroup level. Emerg Infect Dis. 2024;30:1631–41. 10.3201/eid3008.240209 39043393 PMC11286072

[4] Patel H, Monzon S, Varona S, Espinosa-Carrasco J, Garcia MU, Heuer ML, et al. nf-core/viralrecon: v2.6.0-Rhodium Raccoon; 2023. [cited 2024 Aug 1]. https://zenodo.org/records/7764938

[5] Aksamentov I, Roemer C, Hodcroft EB, Neher RA. Nextclade: clade assignment, mutation calling and quality control for viral genomes. J Open Source Softw. 2021;6:3773. 10.21105/joss.03773

[6] Hadfield J, Megill C, Bell SM, Huddleston J, Potter B, Callender C, et al. Nextstrain: real-time tracking of pathogen evolution. Bioinformatics. 2018;34:4121–3. 10.1093/bioinformatics/bty407 29790939 PMC6247931

[7] Huddleston J, Hadfield J, Sibley TR, Lee J, Fay K, Ilcisin M, et al. Augur: a bioinformatics toolkit for phylogenetic analyses of human pathogens. J Open Source Softw. 2021;6:2906. 10.21105/joss.02906 34189396 PMC8237802

[8] Katoh K, Standley DM. MAFFT multiple sequence alignment software version 7: improvements in performance and usability. Mol Biol Evol. 2013;30:772–80. 10.1093/molbev/mst010 23329690 PMC3603318

[9] Minh BQ, Schmidt HA, Chernomor O, Schrempf D, Woodhams MD, von Haeseler A, et al. IQ-TREE 2: new models and efficient methods for phylogenetic inference in the genomic era. Mol Biol Evol. 2020;37:1530–4. 10.1093/molbev/msaa015 32011700 PMC7182206

[10] Kumar S, Suleski M, Craig JM, Kasprowicz AE, Sanderford M, Li M, et al. TimeTree 5: an expanded resource for species divergence times. Mol Biol Evol. 2022;39:174. 10.1093/molbev/msac174 35932227 PMC9400175

[11] Seemann T. SNP-distances. 2018 [cited 2024 Aug 1]. https://github.com/tseemann/snp-dists

[12] Paradis E, Schliep K. ape 5.0: an environment for modern phylogenetics and evolutionary analyses in R. Bioinformatics. 2019;35:526–8. 10.1093/bioinformatics/bty633 30016406

[13] Langedijk AC, Harding ER, Konya B, Vrancken B, Lebbink RJ, Evers A, et al. A systematic review on global RSV genetic data: Identification of knowledge gaps. Rev Med Virol. 2022;32:e2284. 10.1002/rmv.2284 34543489 PMC9285027

[14] Havers FP, Whitaker M, Melgar M, Chatwani B, Chai SJ, Alden NB, et al.; RSV-NET Surveillance Team. Characteristics and outcomes among adults aged ≥60 years hospitalized with laboratory-confirmed respiratory syncytial virus—RSV-NET, 12 states, July 2022–June 2023. MMWR Morb Mortal Wkly Rep. 2023;72:1075–82. 10.15585/mmwr.mm7240a1 37796742 PMC10564327

