# Supplementary material for: Genomic Epidemiology of Human Respiratory Syncytial Virus, Minnesota, USA, July 2023–February 2024
Source: Emerg Infect Dis. 2024 Nov;30(11):2414–8. doi: 10.3201/eid3011.241000 (PMC11521169; doi:10.3201/eid3011.241000)
Supplement: Appendix — Additional information for genomic epidemiology of human respiratory syncytial virus, Minnesota, USA, July 2023–February 2024. [file 24-1000-Techapp-s1.pdf]

Article DOI: http://doi.org/10.3201/eid3011.241000

EID cannot ensure accessibility for supplementary materials supplied by authors. Readers who have difficulty accessing supplementary content should contact the authors for assistance.

Genomic Epidemiology of Human Respiratory Syncytial Virus, Minnesota, USA, July 2023–February 2024

Appendix

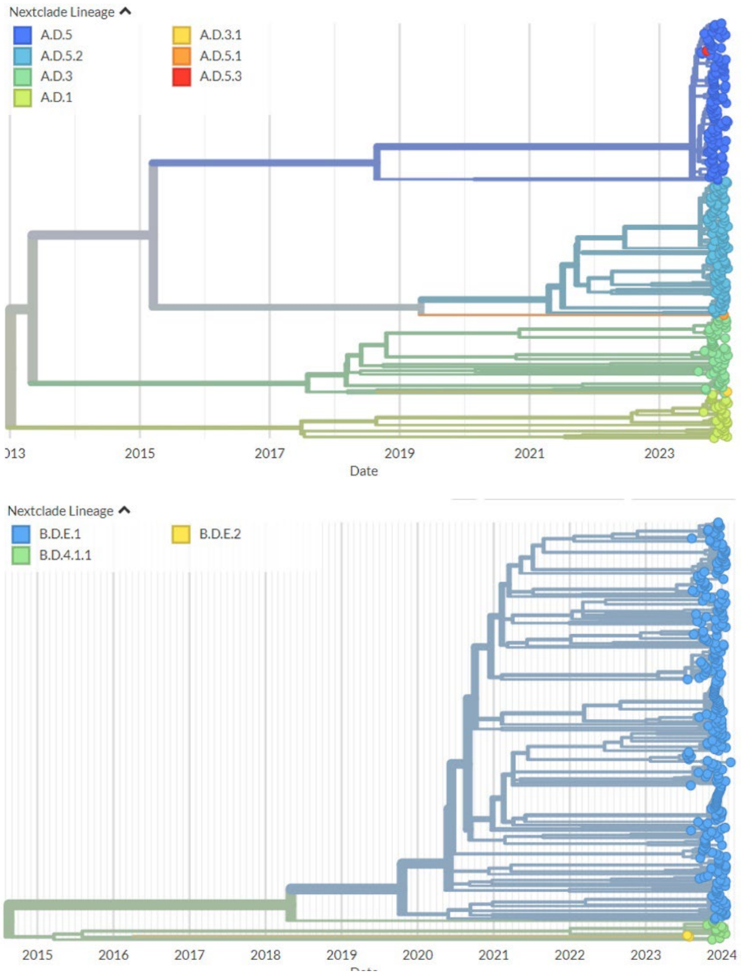

Appendix Figure. Phylogenetic trees shown in Figures 1 and 2 in the main text, annotated in Nextstrain software with a scale of estimated divergence dates by year. Colors denote the lineage classifications of viral genomes as described by Goya et al. (*1*). Estimated times of lineages' most recent common ancestors (tMRCAs) and corresponding 95% confidence intervals are shown in Table 1 in the main text.

Reference

1. Goya S, Ruis C, Neher RA, Meijer A, Aziz A, Hinrichs AS, et al. Standardized phylogenetic classification of human respiratory syncytial virus below the subgroup level. Emerg Infect Dis. 2024;30:1631–41. PubMed https://doi.org/10.3201/eid3008.240209
